# Scientific rationale for the use of α2A-adrenoceptor agonists in treating neuroinflammatory cognitive disorders

**DOI:** 10.1038/s41380-023-02057-4

**Published:** 2023-04-07

**Authors:** Amy F. T. Arnsten, Yumiko Ishizawa, Zhongcong Xie

**Affiliations:** 1 Department Neuroscience, Yale University School of Medicine, New Haven, CT 056510 USA; 2 Department Anesthesiology, Critical Care and Pain Medicine, Massachusetts General Hospital, Harvard Medical School, Boston, MA 02114 USA

**Keywords:** Neuroscience, Diseases

## Abstract

Neuroinflammatory disorders preferentially impair the higher cognitive and executive functions of the prefrontal cortex (PFC). This includes such challenging disorders as delirium, perioperative neurocognitive disorder, and the sustained cognitive deficits from “long-COVID” or traumatic brain injury. There are no FDA-approved treatments for these symptoms; thus, understanding their etiology is important for generating therapeutic strategies. The current review describes the molecular rationale for why PFC circuits are especially vulnerable to inflammation, and how α2A-adrenoceptor (α2A-AR) actions throughout the nervous and immune systems can benefit the circuits in PFC needed for higher cognition. The layer III circuits in the dorsolateral PFC (dlPFC) that generate and sustain the mental representations needed for higher cognition have unusual neurotransmission and neuromodulation. They are wholly dependent on NMDAR neurotransmission, with little AMPAR contribution, and thus are especially vulnerable to kynurenic acid inflammatory signaling which blocks NMDAR. Layer III dlPFC spines also have unusual neuromodulation, with cAMP magnification of calcium signaling in spines, which opens nearby potassium channels to rapidly weaken connectivity and reduce neuronal firing. This process must be tightly regulated, e.g. by mGluR3 or α2A-AR on spines, to prevent loss of firing. However, the production of GCPII inflammatory signaling reduces mGluR3 actions and markedly diminishes dlPFC network firing. Both basic and clinical studies show that α2A-AR agonists such as guanfacine can restore dlPFC network firing and cognitive function, through direct actions in the dlPFC, but also by reducing the activity of stress-related circuits, e.g. in the locus coeruleus and amygdala, and by having anti-inflammatory actions in the immune system. This information is particularly timely, as guanfacine is currently the focus of large clinical trials for the treatment of delirium, and in open label studies for the treatment of cognitive deficits from long-COVID.

## Introduction

Stress and inflammation can profoundly alter the nervous and immune systems, creating long-term impairments in cognitive functioning. In particular, the cognitive functions of the prefrontal cortex (PFC) are impaired in a wide range of neuroinflammatory disorders, including in Alzheimer’s disease (AD) [1], traumatic brain injury (TBI) [2], and perioperative neurocognitive disorder (PND) [3]. The PFC subserves abstract reasoning and the executive functions, where deficits can be remarkably debilitating, interfering with the ability to work or care for families [4]. There is a great need for treatment, especially in the wake of COVID-19 infection, where cognitive deficits from “long-COVID” are prevalent [5]. However, there are no FDA-approved medications for treating neuroinflammatory cognitive disorders.

New research has shown that the recently evolved circuits in the primate PFC have unusual molecular needs that support higher cognitive operations, but render these circuits particularly vulnerable to disruption from neuroinflammation [6]. In particular, these circuits express the molecular machinery for cAMP to magnify calcium signaling to help sustain prolonged neuron firing needed to represent information without sensory stimulation. However, magnified cAMP-calcium signaling is readily dysregulated by stress and inflammation, leading to atrophy, tau phosphorylation and impaired PFC cognitive functioning. Thus, medications that can restore regulation of cAMP-calcium signaling may be especially helpful in restoring cognitive abilities.

As will be described in detail below, research has shown that the selective noradrenergic α2A-adrenoceptor (α2A-AR) agonist, guanfacine, can strengthen PFC network connections and improve PFC cognitive functioning by regulating cAMP-calcium signaling [7]. Based on this research, extended release guanfacine was approved by the FDA in 2009 for the treatment of Attention Deficit Hyperactivity Disorder, which is characterized by PFC deficits. Guanfacine is also being used off-label and/or being tested to treat cognitive deficits in a variety of neuroinflammatory disorders including traumatic brain injury (TBI), delirium (post-anesthetic or infectious), and cognitive deficits from long-COVID (“brain fog”). However, the scientific bases for guanfacine’s actions in these disorders are generally not understood, as this research has arisen from multiple, and often disparate, disciplines. While many scientists and physicians are aware of how stimulation of presynaptic α2A-AR autoreceptors reduces NE release from the sympathetic nervous system [8], they are often unaware of important, post-synaptic α2A-AR actions in brain that are especially relevant to cognitive functioning. The current review describes new data on why PFC circuits are so vulnerable to neuroinflammation, and how α2A-AR stimulation can coordinate a “safety signal” across the neuroaxis and the immune system that helps to restore normative brain and inflammatory functioning needed for healthy cognition.

## The functions of the prefrontal cortex

The PFC enlarges greatly in primates and is topographically organized, with the dorsal and lateral regions (dlPFC) subserving cognitive functioning, and the ventral (i.e. orbital) and medial regions (vmPFC) regulating emotional states [4, 9–11]. These areas are tightly interconnected, e.g. with adaptable evaluations of reward values being conveyed from the vmPFC to the dlPFC, and the dlPFC providing top-down control of emotion via projections to the vmPFC [12].

dlPFC circuits have the extraordinary ability to generate, sustain, and manipulate representations of information in the absence of sensory inputs, the foundation of abstract thought and flexible behavioral responding [13]. These capabilities arise from extensive, recurrent excitatory connections on glutamate synapses in layer III that allow the PFC to sustain information without the need for sensory stimulation [13]. The dlPFC subserves higher order cognitive functions such as working memory, recall memory (but *not* recognition memory, which is more dependent on medial temporal lobe), abstract reasoning, and the executive functions e.g. planning and organization, and the regulation of attention and action [4, 14]. For example, dlPFC networks are needed to effectively divide or focus attention, suppressing interference and noise, and to inhibit inappropriate responses. They contribute to the brain networks that govern complex decision-making, social cognition and self-regulation. In humans, the lateral PFC also generates language, with verbal content generated in the dominant hemisphere, and emotional content generated in the nondominant hemisphere [4]. The most rostral PFC areas in the frontal pole [15] subserve metacognitive functions such as insight and judgment, and metamemory, i.e. remembering to remember. The frontal pole and the dlPFC provide important regulation of emotional states (motivation, anxiety, aggression, depression) through connections with the vmPFC. For example, dlPFC activity and/or synaptic integrity is related to the ability to overcome fatigue [16], stress [17, 18], and depression [19], emphasizing its important, top-down control of emotional state.

## Prefrontal functions are impaired in a large number of neuroinflammatory disorders

PFC functioning is impaired in many neuroinflammatory disorders, even when it is not the primary/initial site of the insult. For example, in rat models, TBI to the posterior cortex impairs PFC function through a number of molecular changes in PFC in response to the distant injury [20–23]. PFC cognitive deficits can range from being relatively mild (although still debilitating), e.g. with executive dysfunction as the primary complaint, to very severe, e.g. profound deficits in delirium. Although delirium involves global deficits in multiple cognitive domains [24], mediated by multiple brain areas, those governed by the PFC can be particularly important to a coherent cognitive state. The following briefly reviews PFC cognitive dysfunction associated with common neuroinflammatory conditions.

### Traumatic brain injury and chronic traumatic encephalopathy

The prevalence of PFC cognitive deficits following mild TBI has been appreciated for decades [25–27], including deficits in executive function as reflected in persistent impairment in performance of the Stroop interference task [28]. TBI also increases risk of depression and/or PTSD [29], a phenomenon also seen in rodents [30]. As described in more detail below, studies in rodents have shown that TBI to the posterior cortex evokes chemical changes in the PFC that impair working memory, even though the initial insult was distant from the PFC [20]. Thus, head injuries to nonfrontal aspects of the head can still result in impaired PFC functioning. Impaired PFC functions are also a major component of chronic traumatic encephalopathy (CTE) [31, 32], where the loss of inhibitory control over aggressive impulses and the increased prevalence of depression have received particular media attention.

### Anesthesia and surgery

Some inhalational anesthetics produce residual cognitive deficits, especially in the elderly and/or young patients with repeated use [33] and reviewed in [34]. Pre-morbid impairments in PFC function (executive function, depression) are a risk factor for post-operative emergence delirium immediately after surgery [35]. In addition, postoperative cognitive disturbances, i.e. PND, can sometimes continue for months. The tasks most commonly used to assess pre- vs. post-operative cognitive performance are those that depend on the functional integrity of the PFC, e.g. tests of working memory, the Trails B test of flexible attention and inhibitory control, and the Stroop interference task [3]. However, recent data suggest that acute exposure to anesthesia alone, in the absence of surgery, did not impair cognition [36, 37], suggesting that the trauma of the surgical procedure itself is a critical interacting factor.

### Hypoxia

The cognitive deficits following hypoxic events are variable based on the cause and duration of the hypoxia, but reviews describe that either acute or chronic hypoxia can impair attention, working/recall memory and executive functioning [38, 39]. Controlled experiments in rats show that chronic exposure to hypoxia impairs working memory in rats, and that it is associated with calcium dysregulation and dendritic spine loss from PFC neurons, i.e. loss of PFC connections [40].

### Cognitive deficits from long-COVID

Patients often describe “brain fog” as a residual and persistent symptom of SARS-CoV-2 infection. It is noteworthy that these cognitive deficits often occur in patients who were never hospitalized with respiratory issues, indicating that they can occur independent of the hypoxia that accompanies severe COVID-19 illness [41]. These cognitive symptoms consistently include impairments in working memory, recall memory, abstract reasoning and the executive abilities, interfering with top-down control of attention, action and emotion [5, 41–43]. The sparing of recognition memory in long-COVID is consistent with the memory functions of the medial temporal lobe being less afflicted compared to operations dependent on the PFC [42]. Depression and anxiety are also common features of long-COVID [41], consistent with reduced regulation of emotion.

### Delirium arising from critical illness

In contrast to long-COVID where cognitive deficits outlast the initial infection, the more severe cognitive deficits of delirium often occur during an active, systemic infection. By definition, delirium involves global deficits in multiple cognitive/attention domains [44]. However, the operations governed by the PFC may be particularly important for calming an agitated individual, and for effective communication [45]. The elderly are at much greater risk of delirium [24], and this may be related to the dysregulation of calcium signaling and rise in neuroinflammation in the aged PFC [6, 46, 47]. As described below, the α2A-adrenoceptor agonist, guanfacine, is currently being tested as a treatment for delirium in the context of critical illness, and its ability to reestablish PFC functioning may be an important beneficial component.

## Unusual neurotransmission and neuromodulation renders dlPFC circuits especially vulnerable to neuroinflammation

The following sections describe the unique molecular characteristics of PFC circuits that render them so vulnerable to neuroinflammation, and which make them amenable to guanfacine treatment. Although this is an arena where there is still much to be learned, it is already evident that two major features of layer III dlPFC circuits confer vulnerability: 1) they have unique neurotransmission [48, 49] that makes them especially vulnerable to kynurenine inflammatory signaling, and 2) they have unusual neuromodulation, where feedforward cAMP-calcium signaling opens K^+^ channels to reduce neuronal firing, a process that can lead to toxic actions when regulation is weakened by inflammatory changes [6].

### PFC circuits have unique neurotransmission that would be especially vulnerable to kynurenic acid inflammatory signaling

#### Unusual neurotransmission in dlPFC

**Fig. 1:**
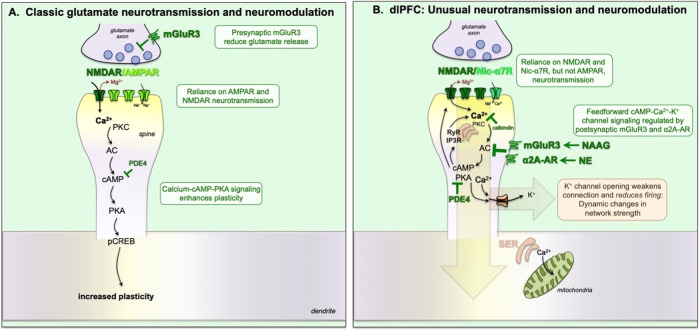
Schematic illustration of neurotransmission and neuromodulation in a classic vs. dlPFC glutamate synapse. **A** In a classic glutamate synapse, neurotransmission depends on AMPA receptors, which depolarize the postsynaptic membrane to eject Mg^2+^ from the NMDAR pore and allow NMDAR neurotransmission (i.e.”permissive” actions). The calcium entry through NMDAR can drive cAMP-PKA signaling to increase neuroplasticity and strengthen connections. cAMP is catabolized by PDE4s, which reduce memory formation. In traditional glutamate synapses, mGluR3 are often *presynaptic*, where they reduce glutamate release. **B** In contrast to traditional glutamate circuits which depend heavily on AMPAR, layer III dlPFC circuits have little AMPAR dependence, and instead rely most heavily on NMDAR and nic-α7R, which reside within the glutamate synapse and help to depolarize the postsynaptic membrane, needed to eject the Mg^2+^ for NMDAR actions. Depolarization may also be supported by high levels of calcium, including cAMP-PKA magnification of calcium release from the smooth endoplasmic reticulum (SER), shown in pink. Calcium in turn drives cAMP production, leading to feedforward signaling. Higher levels of cAMP-PKA-calcium signaling open K^+^ channels to weaken connectivity and have dynamic changes in synaptic strength, reducing firing under conditions of high cAMP-calcium signaling (see Fig. 2). Under healthy conditions, feedforward cAMP-PKA-calcium-K^+^ signaling is tightly regulated, by *postsynaptic* mGluR3 and α2A-AR inhibition of cAMP synthesis, by PDE4 catabolism of cAMP, and calbindin binding of cytosolic calcium.

Traditional glutamate neurotransmission requires AMPAR stimulation, which depolarizes the synaptic membrane, relieving the magnesium block of the NMDAR channel, and thus permitting and/or facilitating NMDAR neurotransmission [50, 51] (Fig. 1A). Thus, the depolarizing effects of AMPAR are considered “permissive” for NMDAR actions. Most AMPAR flux sodium but not calcium, and have rapid on/off characteristics that are especially amenable to coding sensory events. However, the synapses in layer III of dlPFC have very different neurotransmission, likely reflecting their need to sustain the neuronal firing of recurrent excitatory circuits for many seconds. As schematized in Fig. 1B, these neurons depend on NMDAR, with surprisingly little contributions of AMPAR stimulation [48]. Instead, the permissive excitation of the synaptic membrane is performed by acetylcholine stimulation of nic-α7R, which reside within the glutamate synapse [49]. The reliance on acetylcholine may be particularly important for coordinating dlPFC actions with arousal state, as acetylcholine is released during waking (conscious), but not deep sleep (unconscious). Studies in monkeys have shown that local blockade of NMDAR or nic-α7R markedly reduces the dlPFC neuronal firing needed for working memory, while systemic administration of NMDAR blockers impairs cognitive performance in monkeys and humans [48, 52, 53], and that cholinergic depletion of the dlPFC is devastating to working memory [54], demonstrating the importance of both NMDAR and cholinergic receptors to higher cognitive abilities.

#### Kynurenine inflammatory signaling

**Fig. 2:**
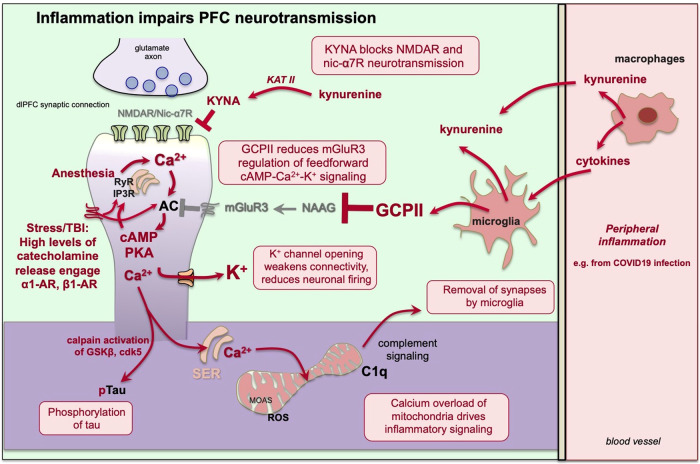
The unusual molecular properties of primate layer III dlPFC make it particularly vulnerable to neuroinflammation, including loss of neuronal firing, loss of synapses, and tau phosphorylation. Kynurenine is made and released under inflammatory conditions and metabolized to KYNA in brain, where it blocks NMDAR and nic-α7R, which would greatly reduce dlPFC neurotransmission. Reactive microglia and astroctyes make and release GCPII, which catabolizes NAAG and thus reduces mGluR3 regulation of cAMP synthesis. There also can be loss of PDE4s and calbindin (not shown, see text). Psychological and physiological stress release high levels of catecholamines in PFC, that drive cAMP-PKA-calcium signaling; anesthesia can also increase calcium release from the SER. Dysregulated cAMP-PKA-calcium signaling leads to: 1) loss of neuronal firing via opening of K^+^ channels, 2) calcium overload of mitochondria, initiating inflammatory signaling including signals to microglia to remove synapses, and 3) tau hyperphosphorylation, e.g. through calpain 2 cleavage and activation of GSK3β and cdk5, the primary kinases that phosphorylate tau.

Under conditions of inflammation, there are large increases in the production of kynurenine. Kynurenine is metabolized from tryptophan in the immune system (e.g. by macrophages [55]) and in microglia [56], and may provide a source of cellular energy (nicotinamide adenine dinucleotide, i.e. NAD + ) [57]. Plasma kynurenine is actively taken up into brain [58], and thus peripheral insults can have indirect but large effects on brain functioning. Kynurenine can be further metabolized to quinolinic acid (QUIN), which kills neurons, or to kynurenic acid (KYNA) (Fig. 2), which blocks both NMDAR and nic-α7R [59]. (As charged metabolites (e.g. KYNA, QUIN) do not normally cross the blood-brain barrier, it can be difficult to glean possible CNS actions from serum levels of metabolites). Although neuronal apoptosis induced by QUIN may occur in some extreme conditions as described below, many chronic neuroinflammatory disorders with pronounced cognitive deficits are not associated with neuron death. In these disorders, high levels of KYNA may be responsible for cognitive deficits through KYNA blockade of NMDAR and nic-α7R. As the higher cognitive circuits of the dlPFC depend on these two receptors for neurotransmission, KYNA may disproportionately impair dlPFC functioning, producing the patterns of dlPFC cognitive deficits described above. In support of this hypothesis, inhibition of KYNA production in brain improved working memory in aged monkeys [60].

#### Neuroinflammatory disorders associated with elevated kynurenine signaling

Increased plasma kynurenine and/or KYNA levels in brain or CSF have been reported in a number of neuroinflammatory disorders, and with advancing age in humans [61]. The COVID-19 pandemic has brought increased attention to this mechanism, as infection by SARS-CoV-2 greatly increases plasma kynurenine levels [62–64]. Particularly relevant to the current review, cognitive deficits associated with “long-COVID” correlate with continued elevations of kynurenine in blood [65]. Assays of the brains of patients who have died from COVID-19 show large increases in KYNA [66], consistent with uptake and further metabolism in brain. It is likely that these findings with SARS-CoV-2 generalize to other types of infection that induce cognitive changes, as levels of delirium during critical illness also correlate with plasma kynurenine levels [67]. Elevated kynurenine signaling may also be related to post-surgical cognitive deficits, as presurgical elevations in plasma kynurenine correlated with post-surgical deficits in executive function and memory [68]. TBI is also associated with elevated kynurenine in patients and in animal models [69]. High levels of QUIN in CSF during the initial days after the injury are associated with more serious outcomes, including death [70]. However, there has been less research on CSF levels of kynurenine and its metabolites in chronic cognitive disorders associated with TBI where there is little evidence of apoptotic cell death.

### PFC circuits have unique neuromodulation that magnifies toxic calcium actions and reduces neuronal firing under inflammatory conditions

#### Unusual neuromodulation in dlPFC

In addition to atypical neurotransmission, layer III pyramidal cells in the dlPFC also have unusual neuromodulation (Fig. 1B), where magnified calcium signaling is needed to support persistent neuronal firing, but high levels open potassium (K^+^) channels that markedly reduce neuronal firing, creating a narrow inverted-U [6]. These include cAMP opening of HCN and Slack channels [71, 72], PKA opening of KCNQ2 channels [46, 73, 74], and calcium opening of SK channels (Arnsten, unpublished). This differs from traditional neuromodulatory actions, where cAMP-calcium signaling produces a more uniform enhancement in neurotransmission [51] and enhances neuroplasticity [75, 76] (Fig. 1A).

**Fig. 3:**
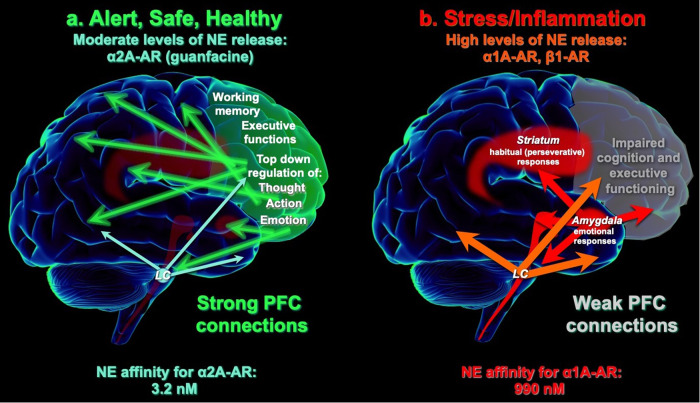
NE acts as a neurochemical switch, determining whether recently evolved or primitive brain circuits govern behavior. **A** Under nonstressful, healthy conditions, moderate levels of NE release engage high affinity α2A-ARs, strengthening the PFC and weakening the amygdala. Thus, there is strong top-down control of attention, action and emotion. **B** Under conditions of physiological or psychological stress, high levels of NE release activate low affinity α1-AR and β-AR, which weaken the PFC and strengthen the amygdala, switching the brain into a more primitive state. Treatment with the α2A-AR agonist, guanfacine, can transition the brain back into a regulated state.

As schematized in Fig. 1B, layer III dlPFC synapses express the molecular machinery for cAMP-PKA signaling to magnify calcium within the cytosol of layer III spines, including increasing internal calcium release from the smooth endoplasmic reticulum (SER), and by phosphorylating NMDAR and voltage-gated calcium channels to increase calcium entry. Calcium in turn promotes cAMP formation, leading to feedforward signaling. Layer III dlPFC spines also express a number of K^+^ channels that are opened by cAMP, PKA or calcium. Moderate levels of cAMP-calcium-K^+^ channel opening allows dynamic changes in network strength (Fig. 1B), as well as negative feedback to prevent seizures [6, 77], providing flexible, “top-down control” of our thoughts, actions and emotions when we feel safe, alert and interested (Fig. 3A). However, high levels of cAMP-calcium-K^+^ signaling, e.g. during stress and/or inflammation, functionally disconnects dlPFC synapses, greatly reducing neuronal firing (Fig. 2). This mechanism allows the dlPFC to be rapidly disconnected and silenced during an uncontrollable stressor, switching control of behavior to more primitive brain circuits that mediate habitual and emotional responding (Fig. 3B). This would have survival value in many dangerous conditions, e.g. being cut off on the highway while driving, but is detrimental when higher cognitive functions are needed to thrive [78, 79].

Under conditions of psychological or physiological stress, high levels of norepinephrine (NE) are released in the PFC [80, 81] (Fig. 2). For example, studies in rats have shown that high levels of catecholamines are released in the PFC with traumatic brain injury, even though the concussion occurs at a large distance from the PFC [82]. High levels of NE engage low affinity receptors (e.g. α1A-AR) that drive calcium-cAMP opening of K^+^ channels (Fig. 2), rapidly taking the PFC “off-line” [83, 84] (Fig. 3B). α1-AR expression is also increased in the rat mPFC following TBI [22].

Under healthy conditions, feedforward cAMP-calcium signaling is regulated by post-synaptic mGluR3 and α2A-AR (Fig. 1B). These receptors are localized on layer III spines, and they inhibit cAMP-calcium opening of K^+^ channels, strengthening network connectivity and enhancing the dlPFC neuronal firing needed for higher cognition. mGluR3 are primarily post-synaptic in the primate dlPFC, and are not only stimulated by glutamate, but by N-acetylaspartylglutamate (NAAG), which is co-released with glutamate and is selective for mGluR3 [85, 86]. α2A-ARs are often pre-synaptic receptors, but also play a major post-synaptic role in primate dlPFC where they are concentrated on dendritic spines near HCN channels [71]. As α2A-ARs have high affinity for NE (Fig. 3A), they are engaged under nonstress conditions with moderate NE release [87]. As described below, α2A-AR stimulation can also weaken the emotional responding of the amygdala, and regulate the firing of NE neurons in the locus coeruleus (LC), thus helping to promote dlPFC top-down control (Fig. 3A). Feedforward cAMP-calcium signaling is also regulated by the phosphodiesterases (PDE4s) which catabolize cAMP and are anchored to the SER by DISC1 [77], and by the calcium binding protein, calbindin, which is expressed in the cytosol of a subset of layer III dlPFC pyramidal cells [88]. These regulatory mechanisms can restore and strengthen dlPFC function.

#### Neuroinflammation increases calcium-cAMP stress signaling pathways

With chronic psychological stress and/or inflammation, the regulatory pathways that would normally limit or reverse the stress response are weakened, leading to toxic calcium actions such as tau hyperphosphorylation, Aβ generation and removal of synapses (Fig. 2) [6, 89, 90]. High levels of cytosolic calcium activate calpains, which cleave and disinhibit the kinases that hyperphosphorylate tau (Fig. 2) [89]. High levels of calcium release from the SER can also cause calcium overload of mitochondria, leading to further inflammatory events, including complement signaling to microglia to remove synapses (Fig. 2) [90]. The PFC appears to be particularly vulnerable to these actions in rodents, monkeys and humans, which likely relates to its built-in mechanisms to magnify calcium and weaken synaptic connectivity.

Research in aged rhesus monkeys has already revealed at least three ways in which inflammation can dysregulate cAMP-calcium stress signaling (Fig. 2), and there are likely many more. Especially important to primate dlPFC, mGluR3 regulation of cAMP-calcium signaling is greatly reduced by inflammatory increases in glutamate carboxypeptidase II (GCPII) [91, 92], which catabolizes NAAG and thus reduces mGluR3 inhibition of cAMP signaling (Fig. 2). GCPII is synthesized and released by glia, including large increases in GCPII expression by reactive microglia under conditions of inflammation [91, 92]. Physiological studies in aged monkeys show that GCPII markedly lowers the dlPFC neuronal firing needed for higher cognition [86]. PDE4s are also lost with age in primate dlPFC [88, 93], and this may involve MK2 inflammatory signaling unanchoring PDE4 from DISC1 [94]. Finally, calbindin is normally protective against neuronal damage [95], but calbindin levels are lost with early life stress [96], COVID-19 infection [66], or advanced age;[88, 97] the loss of calbindin in the aging dlPFC is selective to layer III pyramidal cells [88], leaving them more vulnerable to calcium toxic actions and tau pathology [98]. The specific molecular mechanisms leading to calbindin loss are not currently known, but would be an important area for future research. Calcium dysregulation can in turn drive more inflammatory signaling, driving vicious cycles [90, 99]. The reduced regulation of cAMP-calcium actions would allow stress signaling to proceed unimpeded, eroding connections and neuronal firing, and building tau pathology within dendrites, increasing risk of future Alzheimer’s disease.

#### Examples of neuroinflammatory disorders with increased calcium-cAMP signaling

A number of neuroinflammatory conditions are associated with calcium dysregulation. For example, anesthetics such as sevoflurane can increase calcium efflux from the SER and induce tau hyperphosphorylation [100–103]. In rodent models, TBI to the posterior cortex causes increased PKA signaling in the PFC that is accompanied by loss of dendritic spines [104]. A similar profile of calcium dysregulation and spine loss is seen in rats exposed to chronic hypoxia [105], where upregulation of GCPII expression contributes to the loss of calcium regulation [106]. Elevated GCPII has been found in mouse models of a variety of disorders, including TBI, ischemia, multiple sclerosis and ALS [107–113]. Calcium dysregulation and tau hyperphosphorylation are also seen in rodent models of repeated head injury [114, 115], consistent with the extensive calcium toxicity and tau pathology seen in human brains with CTE [32, 116]. Finally, SARS-CoV-2 infection greatly increased cAMP-calcium signaling and tau phosphorylation in brain, and was associated with large increases in GCPII expression [66]. Thus, a constellation of elevated GCPII, calcium dysregulation, tau pathology and synapse loss is common to many neuroinflammatory conditions (Fig. 2). It should be noted that GCPII inhibitors are currently under development to treat neuroinflammatory conditions, but existing compounds have relatively poor brain penetration [86, 112, 117].

## Guanfacine beneficial mechanisms

**Fig. 4:**
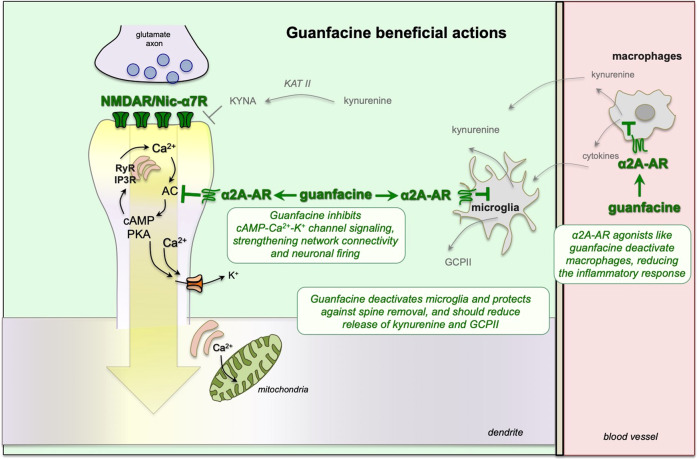
The α2A-AR agonist, guanfacine, restores synaptic connectivity in PFC through both direct and indirect actions. Guanfacine directly strengthens dlPFC connections and neuronal firing by inhibiting cAMP-PKA-calcium-K^+^ signaling in layer III spines. It can also have indirect benefits through its anti-inflammatory actions.

The α2A-AR agonist, guanfacine, is being used off-label and/or tested in a variety of neuroinflammatory cognitive disorders. This section describes both the direct and indirect ways that guanfacine can strengthen PFC cognition and calming effects, summarized in Fig. 4. Although many scientists and physicians are aware of α2A-AR presynaptic actions that reduce catecholamine release e.g. [118], the majority of α2A-ARs are actually post-synaptic in brain [119], including the powerful, post-synaptic effects in PFC that contribute to the calming effects of α2A-AR agonists [7, 71, 120]. Table 1 describes the affinities of commonly used α2-AR agonists for α2-AR subtypes [121, 122]. Guanfacine is the most selective α2A-AR agonist currently available (Table 1), and it is also the least sedating, likely related to its subtype selectivity [123]. In contrast, clonidine and dexmedetomidine have high affinity for all α2-AR subtypes (Table 1), and have prominent sedation. The following section reviews the biological mechanisms through which α2-AR agonists, and guanfacine in particular, may be helpful in treating the cognitive deficits of neuroinflammatory disorders. In addition to traditional presynaptic actions, guanfacine may be especially helpful by restoring regulation of cAMP-calcium-K^+^ signaling in the dlPFC, by weakening the amygdala, and by its anti-inflammatory actions, inhibiting the reactivity of microglia and macrophages, all of which are reviewed below.

**Table 1:** Agonist affinities at α2-AR subtypes.

Agonist	α2A-AR	α2B-AR	α2c-AR
**Kd (nM)**^**a**^			
clonidine	73–84	71	81
dexmeditomidine	18	31	nd
guanfacine	14–31	1850	421
**Ki (nM)**^**b**^			
clonidine	43	106	233
dexmeditomidine	6	18	38
guanfacine	72	1200	2505

^a^from references [114, 115].

^b^from https://en.wikipedia.org/wiki/Alpha-2_adrenergic_receptor citing the following database: Roth BL, Dricol J, PDSP Ki Database:

https://web.archive.org/web/20131108013656/http://pdsp.med.unc.edu/pdsp.php

*nd* not done

### Strengthening and protection of prefrontal cortex

As schematically illustrated in Fig. 4, guanfacine can protect and fortify dlPFC circuits by inhibiting feedforward cAMP-calcium signaling, closing nearby K^+^ channels to strengthen synaptic connectivity and enhance neuronal firing needed for working memory and top-down control [7]. For example, immunoelectron microscopy has demonstrated α2A-AR localized post-synaptically on layer III dendritic spines in the primate dlPFC, next to HCN channels whose open state weakens neuronal firing [71]. Local iontophoresis of guanfacine onto dlPFC neurons enhanced neuronal firing during working memory by inhibiting cAMP signaling [71], and was particularly effective in neurons with dysregulated cAMP-calcium-K^+^ signaling, e.g. due to advancing age [46]. Consistent with the electrophysiological results, either local infusion of guanfacine into the PFC [124–126], or systemic administration of guanfacine, improved a range of cognitive functions in monkeys, promoting flexible regulation of behavior, and reduced distraction and aggression [123, 127–133]. Systemic administration of guanfacine also increased regional cerebral blood flow in the dlPFC as monkeys performed a working memory task [134]. As the dlPFC has direct projections to the LC [135], and indirect projections to regulate the amygdala through the vmPFC [12], restoring dlPFC function may have widespread effects on top-down control.

The enhancing effects of α2A-AR stimulation can also be seen in rat medial PFC, where intraPFC infusion of an α2A-AR agonist improves working memory [126] and reduces anxiety-like behaviors [136]. Studies in rats also show that systemic guanfacine treatment protects the PFC from psychological or physiological stress exposure. Guanfacine pretreatment protects working memory performance from acute stress [137], and also protects working memory and PFC dendritic spines from chronic psychological stress [138] or chronic hypoxia [40, 105], including reducing caspase 3 levels within the PFC [105]. The protection of connections on dendritic spines may be particularly important for guanfacine’s therapeutic effects in stress-related disorders.

### Weakening or regulating the amygdala and locus coeruleus to reduce anxiety/stress

**Fig. 5:**
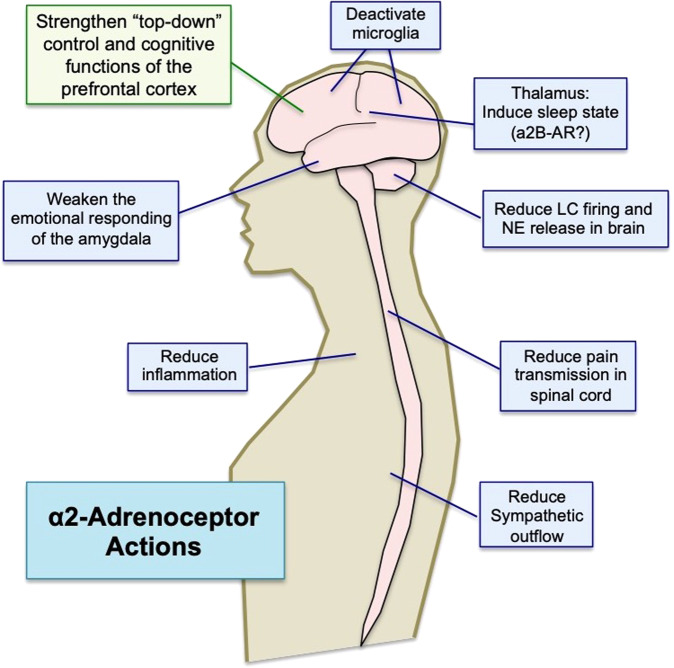
Widespread α2-adrenoceptor actions. α2-adrenoceptor agonists have multiple actions throughout the nervous and immune systems in ways that may benefit the treatment of neuroinflammatory cognitive disorders.

Guanfacine’s calming effects also likely involve additional actions throughout the nervous and immune systems, summarized in Fig. 5. For example, guanfacine may reduce anxiety and emotional reactions by weakening the functions of the amygdala and the extended amygdala (i.e. the bed nucleus of the stria terminalis; BNST). As described above, inhibition of the amygdala may be performed in part from stronger top-down regulation by the PFC. However, there are also strong data showing that α2-AR stimulation directly within the amygdala and/or the BNST reduces fear and anxiety [139], including reduction in plasticity [140], and inhibition of stress-related inputs [141]. Mouse models also show that guanfacine can have anti-depressant like effects through actions within the amygdala [142]. As the amygdala and BNST can activate the LC to initiate a neurochemical stress response [81, 143], deactivation of the amygdala by α2-AR agonists may also have global effects in reducing the stress response.

α2-AR agonists can also have direct effects on the NE system to reduce LC neuronal firing and decrease release from noradrenergic axon terminals. The early, pioneering work of George Aghajanian showed that clonidine reduced LC firing [144]. Both clonidine [145] and guanfacine [146] have been shown to reduce stress-induced increases in catecholamine release. As these high levels of NE release simultaneously impair PFC function [78] and strengthen amygdala via α1-AR and β-AR [147, 148], reducing stress-induced catecholamine release can help to switch the brain back into a regulated state (Fig. 3; [78]). Guanfacine is weaker than clonidine in reducing LC neuronal firing and reducing NE release [149], and recent evidence indicates that chronic guanfacine treatment can actually increase NE release in cortex [150]. These data suggest that reduced NE tone may *not* be the cause of guanfacine’s sustained calming effects.

In summary, the degree of NE release can act as a chemical switch to determine which circuits are in control of our behavior [87]. As shown in Fig. 3A, moderate levels of NE release under nonstress conditions engage high affinity α2A-ARs to strengthen PFC and weaken amygdala, allowing extensive top-down control. In contrast, high levels of NE release during physiological or psychological stress engage low affinity α1-AR and β-AR to weaken PFC and strengthen amygdala (Fig. 3B), switching the brain into a more primitive state [78]. Thus, treatment with the α2A-AR agonist, guanfacine, can help to transition the brain back into a pattern of connectivity that allows more thoughtful, top-down regulation by the PFC. As discussed below, parallel effects may occur in the immune system, with α2-AR stimulation having an anti-inflammatory response.

### Potential sedative effects through the thalamus

α2-AR agonists can produce sedation, which is a side effect when used to treat ADHD, but helpful when used as an anesthetic, e.g., dexmedetomidine [151]. While presynaptic α2A-AR actions appear to contribute to a sleep state [152], they are unlikely the whole story in primates, as the α2A-AR selective agonist, guanfacine, has little sedative actions, while the very sedating dexmedetomidine is a full agonist at the α2B-AR subtype, and only a partial agonist at the α2A-AR and the α2C-AR subtypes (Table 1) [153]. This suggests that the α2B-AR may also provide a large contribution to sedative actions, as least in primates. One possibility is that stimulation of α2B-AR in thalamus produces sedation, as stimulation of post-synaptic α2-AR actions in thalamus induce a sleep state [154], and the thalamus is enriched in α2B-AR [155, 156]. However, this hypothesis has never been actively tested. As the pattern of α2B-AR expression is more extensive in primates than in mice (www.proteinatlas.org/ENSG00000274286-ADRA2B/brain), knockout mouse models may be less useful in determining mechanisms in primates.

### Analgesic actions in the spinal cord

α2-AR agonists have analgesic properties that can be helpful in many medical conditions associated with impaired cognition [157]. At least some of these analgesic actions appear to involve α2-AR stimulation in spinal cord, reducing pain pathway transmission. Descending NE pathways from the LC release NE onto both presynaptic α2-AR on C fiber terminals arising from the dorsal root ganglia, and postsynaptic α2-AR on spinal neurons to reduce the transmission of painful stimuli [158]. For example, dexmedetomidine inhibits the activity of the nociceptive receptors TRPV1 (transient receptor potential vanilloid subtype 1) [159], and the ATP purine receptor P2RX3 [160] in primary sensory neurons in the dorsal root ganglia through an α2A-AR mechanism. Pain may also be diminished through α2-AR anti-inflammatory actions [161] (and see below). It should be noted that in addition to spinal mechanisms, PFC top-down control can also reduce the response to pain [162], and thus α2A-AR actions in PFC may contribute to analgesia as well.

### Anti-inflammatory properties

As schematically illustrated in Fig. 4, α2-AR agonists such as guanfacine, clonidine and dexmedetomidine can have anti-inflammatory actions. In particular, dexmedetomidine has been shown to reduce plasma measures of inflammation during surgery or sepsis [163–166]. The cellular bases for these anti-inflammatory actions are a topic of current research. Most previous research has focused on the roles of β-ARs in regulating the immune system, e.g. as reviewed in [167]. However, dexmedetomidine has been shown to reduce the secretion of the proinflammatory factor, HMGB1 (High mobility group box 1) from LPS-activated macrophages [168]. HMGB1 is a critical proinflammatory factor that has been associated with mortality in sepsis patients. Previous studies have demonstrated that dexmedetomidine can inhibit the secretion of HMGB1 in macrophages induced by lipopolysaccharide (LPS) [168]. Specifically, dexmedetomidine can prevent the translocation of HMGB1 from the nucleus to the cytoplasm and the expression of HMGB1 mRNA. These findings suggest that dexmedetomidine has anti-inflammatory effects and could potentially be used to mitigate the harmful effects of HMGB1 in sepsis. Dexmedetomidine has also been shown to inhibit LPS-induced inflammatory responses by activating PPARγ (peroxisome proliferator-activated receptor gamma) in macrophages [169]. α2A-AR agonists may also reduce inflammation in brain directly, as α2A-AR stimulation causes retraction of reactive microglia [170], which may contribute to guanfacine’s ability to protect dendritic spines from phagocytosis during chronic stress [138] or hypoxia [105]. In rodent models of TBI, dexmedetomidine was shown to reduce monocyte-derived macrophage infiltration into brain [171], and to reduce autophagy and neuroinflammation [172]. α2A-AR are also expressed by retinal ganglion cells, and basic research indicates that α2-AR stimulation in retina is neuroprotective [173]. Thus, α2A-AR agonists may protect against cell death under severe conditions, but may also protect neuronal function needed for higher cognition under more mild conditions by reducing the production of kynurenine and/or GCPII from macrophages and microglia by producing an anti-inflammatory state.

## Guanfacine has beneficial actions in patients

Guanfacine, clonidine and dexmedetomidine are all in widespread clinical use, with the α2A-AR agonist guanfacine being used more prominently for nonsurgical/daytime use due to its less sedating properties. The FDA approved extended release guanfacine (Intuniv™) in 2009 for treating ADHD in children and adolescents, and its approval has been extended to adults with ADHD in Japan [174]. However, guanfacine is also used extensively off-label in a number of conditions that involve PFC dysfunction, including PTSD [175], conduct disorder [176], and autism spectrum disorders [177, 178]. Guanfacine also reduces self-injurious behavior, agitation/aggression and attention/impulse control in patients with Prader-Willi Syndrome [179], similar to its effects in monkeys [132, 180].

In addition to these conditions, guanfacine has been in experimental use, and/or being formally tested, for treating the cognitive disorders discussed in this review. Guanfacine is being used off-label to treat cognitive deficits following TBI, including evidence of enhanced PFC activity and improved working memory with guanfacine treatment [26, 181]. Guanfacine has also been shown to improve attention in patients with contralateral neglect from strokes in the right parietal association cortex [182, 183], and to improve attentional regulation in a patient following encephalomyelitis [184]. Guanfacine, in combination with N-acetylcysteine, is also being used off label to treat cognitive deficits associated with “long-COVID” [185]. It is noteworthy that both COVID-19 infection and TBI increase risk of Alzheimer’s neuropathology, and guanfacine is currently being tested as a potential add-on treatment for early AD [186].

As described above, dexmedetomidine is already in extensive use in the ICU in ventilated patients and in surgery as an anesthetic, and can help diminish delirium in vulnerable subjects [44, 163, 165, 187], including recent data using α2-AR agonists to treat delirium associated with acute COVID-19 infection [188]. However, dexmedetomidine has potent sedating effects that are problematic when the patients need to proceed to a coherent, waking state. Open label data suggest that guanfacine may be particularly useful in this regard, especially in agitated, hyperactive delirium [189–191], where guanfacine’s calming effects may involve many of the mechanisms described above. Intravenous and oral formulations of guanfacine are currently being tested for their effects on delirium from critical illness in large clinical trials (NCT04742673, NCT04578886).

## Summary

In summary, NE stimulation of high affinity α2-ARs normally occurs under conditions of safety, when moderate levels of NE release preferentially engage α2-ARs compared to other adrenoceptors, and are able to coordinate the nervous and immune systems into a nonstressed state. Thus, medications that stimulate α2-ARs receptors may help to restore the brain and body into a healthier configuration. In particular, the ability of the α2A-AR-selective agonist, guanfacine, to restore top-down PFC regulation of behavior with minimal sedation, in addition to its anti-inflammatory properties, provides strong scientific rationale for its daily use in cognitive disorders associated with neuroinflammation.
